# Ruptured abdominal aortic aneurysm and aortocaval fistula: a highly lethal find

**DOI:** 10.1590/1677-5449.202401342

**Published:** 2025-04-14

**Authors:** Walter Mario Ángel Jaramillo, Marby Sharyne Forero Gaviria, Romario Chanci Drago, Natalia Guzmán Arango, Natalia Barrera Cuesta, Andrés Gilberto Giraldo Echeverri, Verónica Lucía Malabet Mejía

**Affiliations:** 1 Hospital Pablo Tobón Uribe, Medellín, Colombia.; 2 Universidad Pontificia Bolivariana, Medellín, Colombia.; 3 Universidad Cooperativa de Colombia, Medellín, Colombia.; 4 Universidad del Norte, Barranquilla, Colombia.

**Keywords:** abdominal aortic aneurysm, aortic ruptura, vascular fistula, inferior caval vein, endovascular aneurysm repair

## Abstract

Within the spectrum of abdominal aortic aneurysm, various complications can arise, including the development of an arteriovenous fistula connecting this major vessel to the infrarenal inferior vena cava. This report presents the case of a 70-year-old man with sudden, intense abdominal pain and syncope, found intraoperatively to have an aortocaval fistula due to a ruptured abdominal aortic aneurysm. The patient underwent emergency care, including fistula closure and placement of a Dacron aortobifemoral graft. Aortocaval fistula is a rare complication of aortic aneurysmal disease, with clinical manifestations varying depending on disease progression, leading to high morbidity and mortality both before and after surgical or interventional treatment.

## INTRODUCTION

The aortocaval fistula (ACF) is an abdominal aortic aneurysm (AAA) complication. This condition is associated with a mortality rate ranging from 22% to 51% and is classified as primary in 80% of cases and secondary in 20%. Among primary cases, 5% to 10% originate from mycotic aneurysms or occur due to Ehlers-Danlos or Marfan syndrome.^1^ In these cases, aortic aneurysms can erode the inferior vena cava (IVC) or iliac veins, leading to an ACF.^2^ The most common ACF location is in the distal abdominal aorta, just above the iliac vein confluence. Most ACFs are asymptomatic, and their clinical presentation depends on the disease’s progression time and whether they are associated with the rupture of a preexisting abdominal aneurysm. When there is no aneurysmal rupture, clinical manifestations include a hyperdynamic hemodynamic state (tachycardia, low diastolic pressure, and cardiac dilation). Additionally, about 80% of patients experience abdominal and/or lumbosacral pain. During physical examination, most present a palpable mass, an audible continuous murmur, and a shiver.^3^ In advanced stages of ACF, the shunting of high-resistance arterial blood into the low-resistance venous system leads to congestive heart failure, commonly with venous hypertension. Additionally, it can affect the digestive and urinary systems, as well as the lower limbs, potentially causing lower gastrointestinal bleeding, hematuria, and lower limb edema.^4^ When aneurysmal rupture occurs, the clinical presentation is consistent with acute abdomen in a patient with hemodynamic instability.^5^

The ideal diagnosis should be confirmed by imaging, with contrast-enhanced abdominal computed tomography (CT) being the method of choice. Radiological findings include aneurysmal dilation of the abdominal aorta, arteriovenous shunt with contrast flow from the aorta to the IVC, and, in some cases, mural thrombus with obstructive effect at the fistula level.^6^

Current treatment strategies include open surgical repair, endovascular repair, or both.^7^ ACF endovascular repair has been increasingly described in the literature, with a variety of techniques and devices available, which are implanted within the abdominal aorta and/or the IVC.^8,9^ Open surgical repair has been the most frequently described treatment and, to date, continues to be considered the treatment of choice. Case complexity and the expertise of the healthcare center professionals are directly related to treatment strategy for this condition.^10^

This case report was approved by the ethics committee of the institution (Pablo Tobón Uribe Hospital).

## PART I – CLINICAL SITUATION

**Figure 1:**
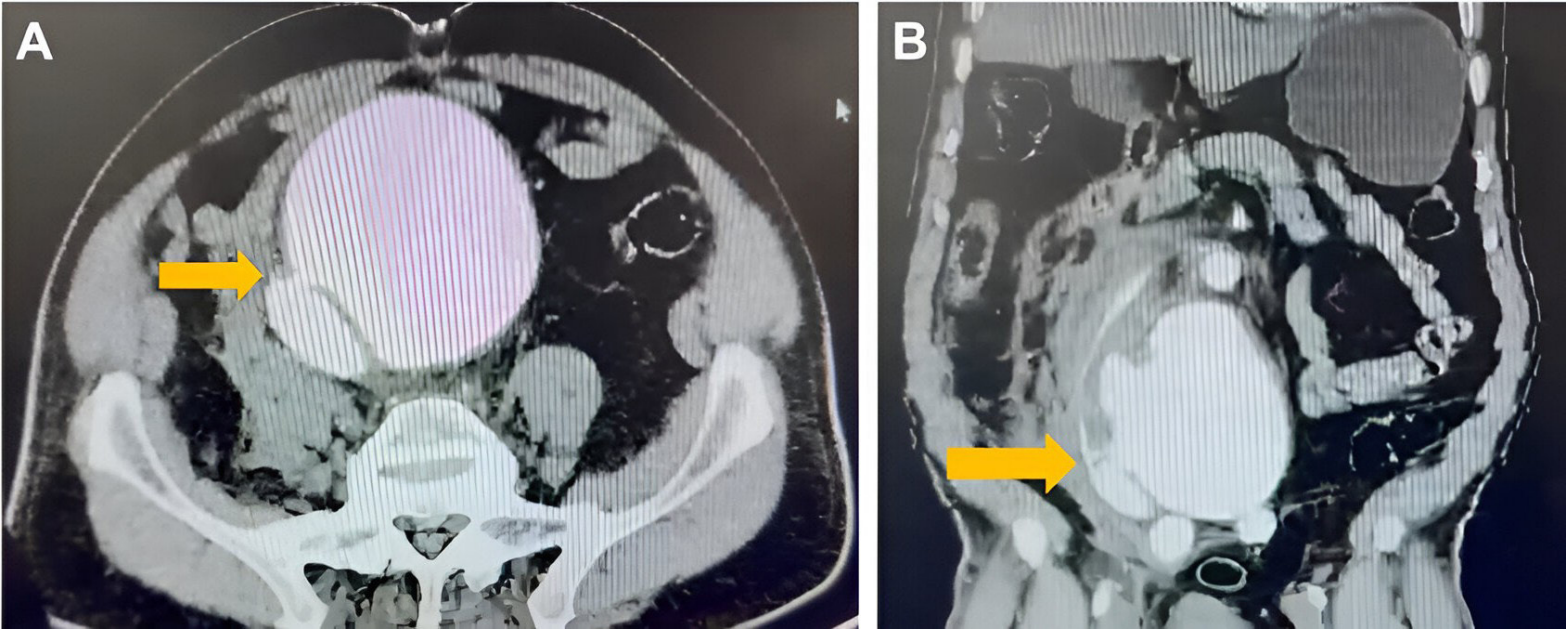
Abdominal computed tomography scan of the patient prior to surgical intervention: (A) axial cut showing the aortocaval arteriovenous fistula (yellow arrow); (B) coronal cut showing the aortocaval arteriovenous fistula (yellow arrow).

The patient was a 70-year-old man, who presented a sudden onset of generalized and intense abdominal pain radiating to the lumbar region, with a syncope episode. He had a history of heavy smoking and no other comorbidities and was admitted to a nearby hospital in poor general condition, diaphoretic, and hypoperfused. During physical examination, marked abdominal distension, a pulsatile mass, and diminished femoral pulses were noted. Arterial blood gases revealed metabolic acidosis and severe hyperlactatemia. An emergency contrast-enhanced abdominal CT scan (Figure 1) was performed, and that was initially interpreted as an abdominal aortic dissection associated with a contained hematoma. Due to this finding, pharmacological control of blood pressure and heart rate were initiated, and the patient was immediately transferred to a higher-level hospital with available vascular surgery.

After clinical evaluation and image review by vascular surgery and radiology, a diagnosis of infrarenal AAA rupture was determined.

Given the diagnosis, the surgical alternatives were questioned: open repair or endovascular technique? However, due to the patient’s rapid clinical deterioration and the lack of immediate availability of trained personnel for endovascular management of the ruptured AAA, an emergency laparotomy was the procedure of choice.

## PART II – WHAT WAS DONE

**Figure 2:**
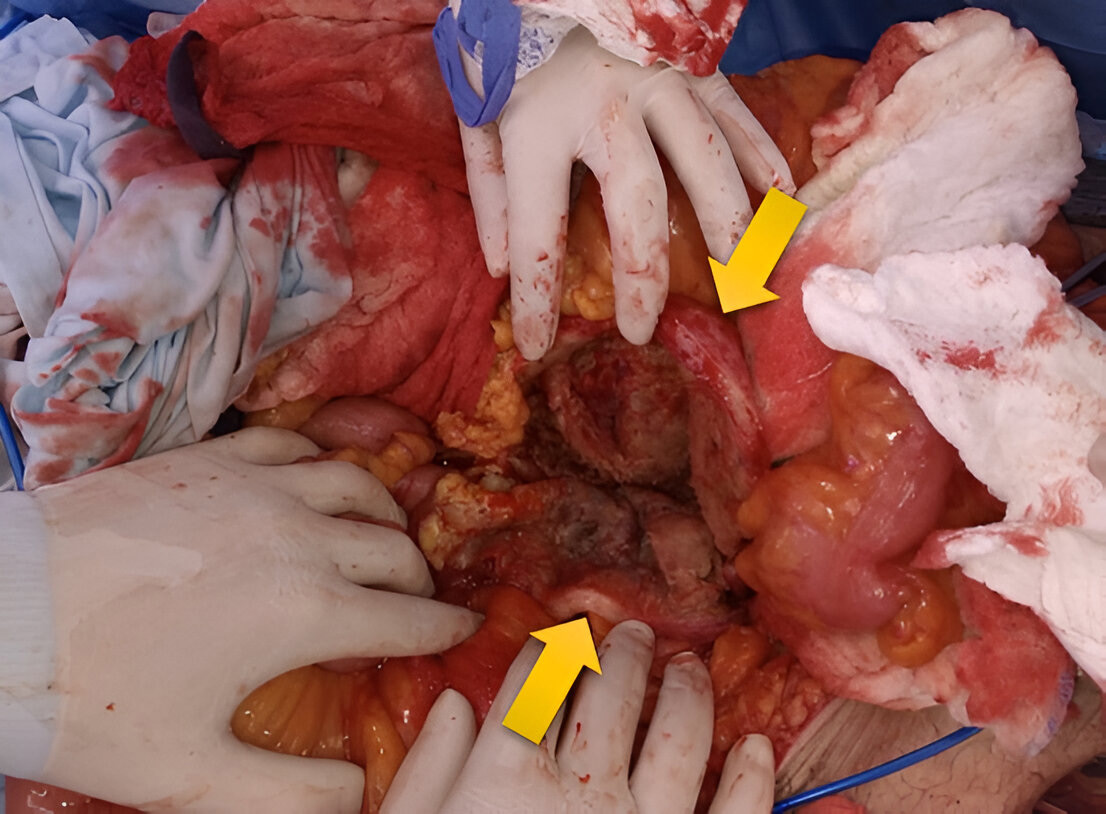
Opening of the aortic aneurysm following vascular control; aortic wall (yellow arrows).

The patient underwent open surgery and, during the procedure, a ruptured infrarenal AAA, approximately 12 cm in diameter, was found. Proximal and distal vascular control was performed, followed by opening the aneurysmal sac (Figure 2). At this point, a 2 cm ACF was also identified, requiring proximal and distal control of the IVC. The ACF was then closed by suturing the venous wall defect with continuous polypropylene sutures. Finally, aortic reconstruction was performed with a Dacron aortofemoral graft.

**Figure 3:**
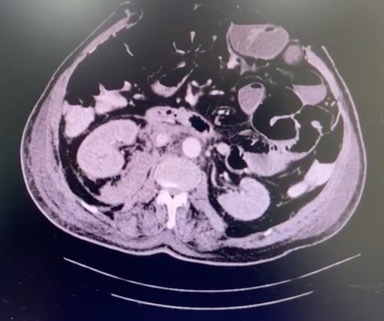
Abdominal computed tomography scan of the patient after surgical intervention, with absence of the aortocaval fistula.

The patient left surgery with an open abdomen and was transferred to the intensive care unit to continue the resuscitation process. During the hospital stay, he progressed well, with a dynamic abdominal wall closure. As a postoperative complication, he developed a urinary tract infection, for which an antibiotic was administered. Recovery was successful, and no complications related to the surgical technique were observed. Postoperatively, a contrast-enhanced abdominal CT scan was performed to evaluate differential diagnoses due to postoperative ileus. No complications related to the surgery were reported, and no ACF was identified (Figure 3).

## DISCUSSION

ACFs are rare complications of AAAs, with high morbidity and mortality rates, significantly affecting multiple body systems. Early diagnosis, treatment, and correction of these fistulas pose a significant challenge.

The classic surgical treatment for ACF focuses on vascular control of the aneurysm, followed by direct closure of the arteriovenous communication from the aneurysmal sac.^11^ Treatment options include open surgery and endovascular treatment,^12,13^ which are elected based on clinical presentation, comorbidities, surgical anatomy, and patient stability in the operating room.

Open surgery remains widely used, with a high success rate, low reintervention rates, and no significant increase in postoperative mortality compared to endovascular treatment. However, it is associated with longer hospital stays and higher morbidity.^7,14^ Conversely, endovascular treatment offers an attractive therapeutic alternative to open surgery, presenting satisfactory results, such as less blood loss.^15^ However, the number of patients treated via the latest approach remains low, and long-term follow-up data are insufficient. Additionally, it presents complications such as endograft thrombosis, endoleaks, among others.^15,16^

However, endovascular repair is the preferred method for treating ACF,^17^ as it presents lower postoperative complication rates compared to open surgery.^18^ When available, endovascular repair is the method of choice,^19^ especially in multimorbid patients with favorable anatomy. Conversely, to avoid follow-up and reinterventions often required by endovascular treatment, open surgery is preferred in young, healthy patients or those with unfavorable anatomy.^20^

## CONCLUSION

ACF is a very rare complication among patients with AAA. Its clinical presentation varies depending on the presence or absence of aneurysmal rupture and the disease's progression.

Proper pre-surgical imaging and interpretation are essential to avoid incorrect diagnoses and, most importantly, to provide the best treatment strategy for the patient.^16^ Although endovascular treatment is preferred today, open surgery remains a viable option.^20^
