# Supplementary material for: Ruptured abdominal aortic aneurysm and aortocaval fistula: a highly lethal find
Source: J Vasc Bras. 2025 Apr 14;24:e20240134. doi: 10.1590/1677-5449.202401342 (PMC11999540; doi:10.1590/1677-5449.202401342)
Supplement: Translation

# Translation: Aneurisma roto de aorta abdominal y fístula aortocava: un hallazgo altamente letal

**DOI:** 10.1590/1677-5449.202401343

**Authors:** Walter Mario Ángel Jaramillo, Marby Sharyne Forero Gaviria, Romario Chanci Drago, Natalia Guzmán Arango, Natalia Barrera Cuesta, Andrés Gilberto Giraldo Echeverri, Verónica Lucía Malabet Mejía

**Affiliations:** 1 Hospital Pablo Tobón Uribe, Medellín, Colombia.; 2 Universidad Pontificia Bolivariana, Medellín, Colombia.; 3 Universidad Cooperativa de Colombia, Medellín, Colombia.; 4 Universidad del Norte, Barranquilla, Colombia.

**Keywords:** aneurisma de la aorta abdominal, rotura de la aorta, fístula vascular, vena cava inferior, reparación endovascular de aneurismas

## Abstract

Dentro del espectro de aneurisma de aorta abdominal, se presentan diversas complicaciones asociadas a esta patología, entre ellas el desarrollo de una fístula arteriovenosa que comunica este gran vaso con la vena cava inferior a nivel infrarrenal. En este reporte, se presenta el caso de un hombre de 70 años con dolor abdominal súbito intenso asociado a síncope, el cual tuvo un hallazgo intraoperatorio de fístula aortocava en el contexto de un aneurisma de aorta abdominal roto. El paciente fue manejado de forma emergente, realizándose el cierre de la fístula y la colocación de un injerto aortobifemoral de dacrón. La fístula aortocava es una complicación rara de la patología aneurismática aórtica, cuyas manifestaciones clínicas varían según el tiempo de evolución de la enfermedad, lo que conlleva una elevada morbimortalidad antes y después de su tratamiento quirúrgico y/o intervencionista.

## INTRODUCCIÓN

La fístula aortocava (FAC) es una de las complicaciones del aneurisma de aorta abdominal (AAA). Esta patología se asocia con una mortalidad que oscila entre el 22% y el 51%, clasificándose como primaria en el 80% de los casos y secundaria en el 20%. Dentro del subtipo primario, entre el 5% y el 10% de los casos se origina a partir de aneurismas micóticos o en el contexto de un síndrome de Ehlers-Danlos o síndrome de Marfan^1^ . En estos casos, los aneurismas aórticos pueden erosionar la vena cava inferior (VCI) o las venas ilíacas, provocando así una FAC^2^ . La localización más frecuente de la FAC es a nivel de la aorta abdominal distal, justo por encima de la confluencia de las venas ilíacas. Casi todas las FAC son asintomáticas, y su manifestación clínica dependerá exclusivamente del tiempo de evolución de la enfermedad y su asociación, o no, con la rotura de un aneurisma abdominal preexistente. Cuando no existe rotura aneurismática, las manifestaciones clínicas incluyen un estado hemodinámico hiperdinámico (taquicardia, presión diastólica baja y dilatación cardiaca). Además, se reporta que aproximadamente el 80% de los pacientes experimentan dolor abdominal y/o lumbosacro. En el examen físico, la mayoría presenta una masa palpable, soplo continuo audible y frémito^3^ . En estadios más avanzados de la FAC, debido al paso de flujo sanguíneo arterial de alta resistencia al sistema venoso de baja resistencia, se presenta también insuficiencia cardiaca congestiva, siendo común el desarrollo de hipertensión venosa. Adicionalmente, puede afectar el aparato digestivo, el aparato urinario y las extremidades inferiores, lo que puede resultar en hemorragia digestiva baja, hematuria y edema de miembros inferiores^4^ . Cuando existe rotura aneurismática, la presentación clínica configura un abdomen agudo en un paciente con inestabilidad hemodinámica^5^ .

El diagnóstico ideal incluye la sospecha clínica asociada a la confirmación imagenológica, siendo la tomografía abdominal con contraste el método de elección. Los hallazgos radiológicos incluyen dilatación aneurismática de la aorta abdominal, *shunt* arteriovenoso con flujo de contraste desde la aorta hacia la VCI y, en ocasiones, se evidencia la presencia de trombo mural con efecto obstructivo a nivel de la fístula^6^ .

Las estrategias de tratamiento actual incluyen reparación quirúrgica abierta, endovascular o una combinación de ambas^7^ . La reparación endovascular de la FAC se ha descrito cada vez más en la literatura, con una variedad de técnicas y dispositivos disponibles, los cuales se implantan dentro de la aorta abdominal y/o en la VCI^8,9^ . La reparación quirúrgica abierta ha sido la modalidad de tratamiento descrita con mayor frecuencia y, hasta la fecha, continúa siendo considerada la de elección. La complejidad del caso y la experiencia del centro de atención se relacionan directamente con la estrategia de tratamiento de esta patología^10^ .

El reporte de caso fue aprobado por el comité de ética de la institución (Hospital Pablo Tobón Uribe).

## PARTE I - SITUACIÓN CLÍNICA

El paciente, un hombre de 70 años, presentó un cuadro súbito de dolor abdominal generalizado e intenso, con irradiación hacia la región lumbar, asociado a episodio de síncope. El paciente, con antecedente de ex tabaquismo pesado y sin otras comorbilidades, ingresó a un hospital cercano en malas condiciones generales, diaforético e hipoperfundido. Al examen físico, se observó distensión abdominal marcada, masa pulsátil y disminución de pulsos femorales. Los gases arteriales evidenciaron acidosis metabólica e hiperlactatemia severa. Se realizó, de forma emergente, tomografía de abdomen contrastada (Figura 1), la cual fue interpretada inicialmente como una disección de aorta abdominal asociado a hematoma contenido. Debido a este hallazgo, se decidió iniciar control farmacológico de la presión arterial y la frecuencia cardiaca, y trasladar al paciente de inmediato a un hospital de mayor complejidad con disponibilidad de cirugía vascular.

**Figura 1:** Tomografía axial computarizada del paciente previo a intervención quirúrgica: (A) corte axial en la que se evidencia comunicación arteriovenosa aortocava (flecha amarilla); (B) corte coronal en la que se evidencia comunicación arteriovenosa aortocava (flecha amarilla).

Posteriormente, tras la valoración clínica y la revisión de imagen por cirugía vascular y radiología, se realizó el diagnóstico de rotura de aneurisma de aorta abdominal infrarrenal.

Dado el diagnóstico establecido, se pone en cuestión las posibles alternativas quirúrgicas aplicables en el momento: ¿reparación abierta o técnica endovascular? Sin embargo, debido al rápido deterioro clínico del paciente y a la falta de disponibilidad inmediata de personal entrenado en manejo endovascular del AAA roto, se optó por llevar el paciente a una laparotomía emergente.

## PARTE II – LO QUE SE HIZO

El paciente fue sometido a manejo quirúrgico abierto. Durante el procedimiento, se encontró un AAA infrarrenal roto de aproximadamente 12 centímetros de diámetro. Se realizó control vascular proximal y distal a la lesión, y apertura del saco aneurismático (Figura 2). Fue en este punto donde se identificó además una FAC de 2 centímetros de longitud, siendo necesario realizar control proximal y distal de la VCI y, posteriormente, cerrar la FAC mediante cierre de defecto de la pared venosa con sutura contínua de polipropileno. Finalmente, se realizó la reconstrucción aórtica con un injerto aortobifemoral de dacrón.

**Figura 2:** Apertura de aneurisma aórtico posterior a control vascular; pared aórtica (flechas amarillas).

El paciente salió de cirugía con abdomen abierto hacia la unidad de cuidados intensivos para continuar el proceso de reanimación. Durante su estancia hospitalaria, presentó una evolución favorable, logrando desempaquetamiento y cierre dinámico de la pared abdominal. Como complicación postoperatoria, desarrolló una infección del tracto urinario, para lo cual se le administró un esquema antibiótico dirigido. El paciente tuvo una evolución exitosa, y en el seguimiento no se evidenciaron complicaciones asociados a la técnica quirúrgica elegida. En el postoperatorio, se le realizó tomografía de abdomen contrastada de control para evaluar diagnósticos diferenciales debido a un cuadro de íleo postoperatorio, en donde no se reportaron complicaciones relacionadas con la cirugía, y se observó la ausencia de la FAC (Figura 3).

**Figura 3:** Tomografía axial computarizada del paciente posterior a intervención quirúrgica, en la cual no se observa la presencia de fístula aortocava.

## DISCUSIÓN

Las FAC son complicaciones poco frecuentes de los AAA, presentan una alta morbimortalidad e impactan significativamente en múltiples sistemas, siendo el diagnóstico precoz un reto para el examinador. Asimismo, el tratamiento y la corrección de estas fístulas constituyen un desafío.

El tratamiento quirúrgico clásico de la FAC está dirigido al control vascular del aneurisma, seguido por cierre directo de la comunicación arteriovenosa desde el saco aneurismático^11^ . Las opciones de tratamiento incluyen la cirugía abierta y el tratamiento endovascular^12,13^ , las cuales se eligen en función de la forma de presentación clínica, morbilidades, la anatomía quirúrgica y la estabilidad del paciente dentro de la sala de operaciones.

La reparación quirúrgica abierta sigue siendo ampliamente aplicada, con una tasa de éxito alta, asociada con menores tasas de reintervención y sin un aumento significativo de las tasas de mortalidad postoperatoria en comparación con el manejo endovascular. Sin embargo, presenta mayor duración de la estancia hospitalaria y mayor morbilidad^7,14^ . Por otro lado, el manejo endovascular ofrece una alternativa terapéutica atractiva respecto a la cirugía abierta, con resultados satisfactorios, como menor pérdida de sangre^15^ . No obstante, el número de pacientes tratados por esta vía sigue siendo escaso, y el seguimiento a largo plazo es insuficiente. Además, presenta complicaciones como trombosis de la endoprótesis, endofugas, entre otras^15,16^ .

Sin embargo, actualmente, la reparación endovascular es el método preferido para tratar la FAC^17^ , debido a que, comparado con la cirugía abierta, presenta menores tasas de complicaciones^18^ . Se describe que, en caso de disponibilidad, la reparación endovascular es el método de elección^19^ , especialmente en pacientes multicomórbidos y con anatomía favorable. Por otro lado, para evitar el seguimiento y las reintervenciones que a menudo requieren el manejo endovascular, en pacientes jóvenes, saludables o con anatomía no favorable, se prefiere la reparación abierta^20^ .

## CONCLUSIÓN

La FAC es una complicación muy poco frecuente entre los pacientes con AAA. Su presentación clínica varía según la presencia o ausencia de rotura aneurismática y el tiempo de evolución de la enfermedad.

Es fundamental la realización y adecuada interpretación de la imagen prequirúrgica para evitar diagnósticos incorrectos y, sobre todo, para ofrecerle al paciente la mejor estrategia de tratamiento^16^ . Aunque al día de hoy se prefiere la reparación endovascular, la cirugía abierta sigue siendo una opción viable^20^ .
